# The power of pooled analysis: WWARN community reaches a critical mass of data

**DOI:** 10.1186/1475-2875-11-S1-O38

**Published:** 2012-10-15

**Authors:** Philippe J Guerin, Carol H Sibley

**Affiliations:** 1 Centre for Tropical Medicine, University of Oxford, Oxford, OX3 7LE, UK

## 

Early warning of emerging resistance is needed to identify new foci, predict their spread and inform prompt containment action to counter and slow the resistance to antimalarials. The Worldwide Antimalarial Resistance Network (WWARN) works to provide tools to this end.

WWARN has developed systems that transform heterogeneous clinical, pharmacology, molecular and in vitro data from all parts of the world into standardized information that can be analysed and directly compared. Bigger data sets give greater statistical power. The repository currently has 75,000 individual patients’ data (3 million records) - 50% of the published ACT clinical data - and represents the largest compilation of malaria data now available.

ACTs are still highly efficacious in most parts of the world, with the important exception of some specific locations in South East Asia. Therefore, the number of reported clinical failures reported is still small. It is therefore critical to identify and deploy now, efficient methods to track the earliest signs of reduced efficacy. This will require interrogation of very large sets of data to test risk factors associated with failures and work with the community to validate informative early markers.

The current WWARN repository also allows questions of public health relevance to be answered. For example, one can test the consequences of variance in dosing strategies of key ACTs and their effects on clinical efficacy, and early parasitological responses. Outputs from the data analysis also highlight gaps in this retrospective data collection and allow planning for filling those gaps. Prospectively, WWARN is developing research tools and services to support diverse malaria research communities to improve the comparability and quality of data being collected, and to increase the power of pooled analysis.

Presented on behalf of the WWARN community.

